# *Dendrophthoe falcata* (L.f.) Ettingsh. and *Dendrophthoe pentandra* (L.) Miq.: A review of traditional medical uses, phytochemistry, pharmacology, toxicity, and applications

**DOI:** 10.3389/fphar.2023.1096379

**Published:** 2023-02-02

**Authors:** Degang Kong, Lu Wang, Yingshuo Niu, Lingmei Cheng, Bo Sang, Dan Wang, Jinli Tian, Wei Zhao, Xue Liu, Yueru Chen, Fulin Wang, Honglei Zhou, Ruyi Jia

**Affiliations:** ^1^ College of Pharmacy, Shandong University of Traditional Chinese Medicine, Jinan, Shandong, China; ^2^ Jinan Hospital of Traditional Chinese Medicine, Jinan, Shandong, China; ^3^ Jinan Third People’s Hospital, Jinan, Shandong, China; ^4^ The Second Affiliated Hospital of Shandong University of Traditional Chinese Medicine, Jinan, Shandong, China; ^5^ Taian City Central Hospital, Taian, Shandong, China

**Keywords:** traditional uses, phytochemistry, pharmacology, toxicity, applications, Dendrophthoe falcata (L.f.) Ettingsh., Dendrophthoe pentandra (L.) Miq

## Abstract

*Dendrophthoe falcata* (L.f.) Ettingsh. (DF) and *Dendrophthoe pentandra* (L.) Miq. (DP) have been traditionally used for the treatment of various ailments, such as cancer, ulcers, asthma, paralysis, skin diseases, tuberculosis, and menstrual troubles, in the ethnomedicinal systems of India and Indonesia. Currently, the chemical structures of 46 compounds have been elucidated from DF and DP, including flavonoids, triterpenes, tannins, steroids, open-chain aliphatics, benzyl derivates, and cyclic chain derivatives. *In vitro assays* have revealed their anti-tumor and anti-microbial activities. *In vivo* studies have unraveled their pharmacological properties against tumors, depression, fertility disorders, inflammatory responses, and so on. Additionally, their weak toxicity to rats and brine shrimp, as well as their promising applications for pharmaceutical preparations and combined medication, were also revealed. Herein, we not only recapitulated traditional medical uses, phytochemistry, pharmacology, toxicity, and applications of DF and DP but also discussed current research limitations and future perspectives, which are instructive for those interested in them and are committed to advancing parasitic plants to the Frontier of phytomedicine. We highlighted that DF and DP will become promising medical plants rather than being discarded as notorious pests, provided that more and deeper research is undertaken.

## Graphical Abstract

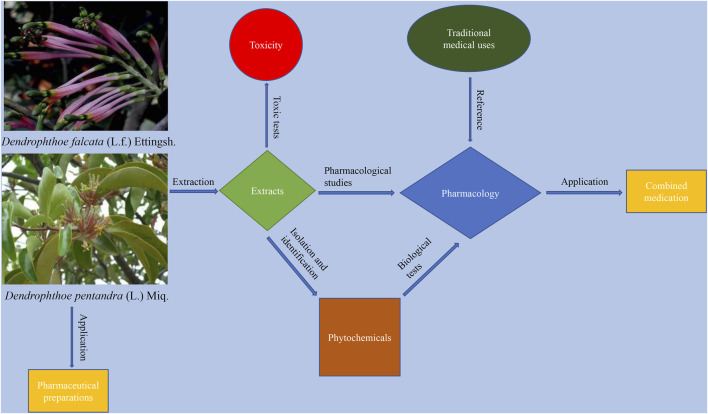

## 1 Introduction

Medical plants are receiving increasingly attention from current researchers as they are available as prescription medications or dietary supplements in most regions (Jalali and Rahbardar, 2022; Jiang et al., 2022). Representative bioactive molecules yielded from natural plants, such as paclitaxel (Klein et al., 2022), progesterone (Prior, 2018), artemisinin (Hanboonkunupakarn et al., 2022), and ginkgolides (Omidkhoda et al., 2019) have been prioritized for clinical treatment at present. As the COVID-19 epidemic spreads around the world, cepharanthine, a botanical drug, has shown more prominent therapeutic effects than the traditional anti-virus drugs we used previously (Chen et al., 2022; Fan et al., 2022). The bioactive compounds are derived from secondary metabolites present in plants, including flavonoids, alkaloids, steroids, terpenes, phenylpropanoids, tannins, and others (Seca and Pinto, 2018; Yan et al., 2022). In general, natural bioactive molecules for clinical treatment are subjected to a series of tests, allowing for structural elucidation, pharmacological assays, and clinical trials. Sometimes, natural ingredients can be deemed as leading compounds for the synthesis of potential candidates for targeted therapies (Gyrdymova and Rubtsova, 2022; Song et al., 2022). Consequently, natural plants provide abundant phytochemicals that can open therapeutic windows for various diseases.

Interestingly, parasitic plants exist as a special class of plants that can share elements, RNA, proteins, and hormone pathways with their hosts (Tennakoon et al., 2011; Westwood and Kim, 2017; Chengala and Menon, 2021; Fàbregas and Fernie, 2022). Sine parasitic plants are present in several plants, they are endowed with a plethora of metabolites and diverse bioactivities (Lim et al., 2017). Mistletoes, in particular, emerge as a taxonomic group for parasitic plants, encompassing several families such as Loranthaceae, Viscaceae, Misodendraceae, Eremolepidaceae, and Santalaceae (Bar-Sela, 2011; Milner et al., 2020). Mistletoes have been traditionally regarded as medical plants with magical powers in view of their omnipotent medicinal properties (Adesina et al., 2013). Birds, as major dispersers, often carry mistletoe seeds to different plants, with the diameter, height, and canopy shape of the hosts being important external factors influencing their choice (Rawsthorne et al., 2011; Rahmad et al., 2014). The seeds can pierce host epidermal tissue, forming an organ called a “haustorium” that allows parasites and hosts to transfer nutrition, carbon, minerals, and water, resulting in a close relationship between them (Jadhav et al., 2005; Adesina et al., 2013; Muche et al., 2022). In taxonomy, parasites can be classified as holoparasites and hemiparasites, depending on whether they have access to photosynthetic resources (Pare and Raynal-Roques, 1999; Zervas et al., 2019). Based on the diverse host parts, parasites can also be called root hemiparasites, root holoparasites, stem parasites, endophytic parasites, and others (Tesitel, 2016).

**FIGURE 1:**
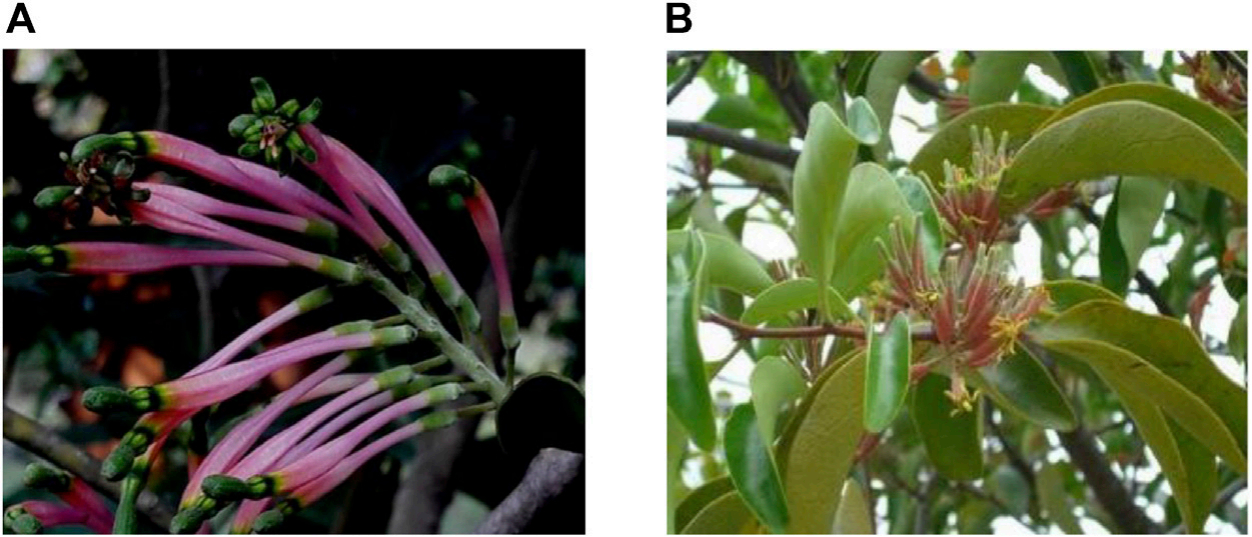
**(A)** *Dendrophthoe falcata* (L.f.) Ettingsh. (Kuramana et al., 2020), **(B)** *Dendrophthoe pentandra* (L.) Miq. (Zainuddin & Sulâ, 2015).

Here, *Dendrophthoe falcata* (L.f.) Ettingsh. (DF) and *Dendrophthoe pentandra* (L.) Miq. (DP) (Loranthaceae) grow throughout the tropics and subtropics as ethnomedicinal plants (Figure 1), captivating our interest as they can be traditionally used for the treatment of common human ailments, such as cancer, ulcers, asthma, paralysis, skin diseases, menstrual troubles, pulmonary tuberculosis, wounds, and so on (Dashora et al., 2011b; Sinoriya et al., 2011; Haque, 2015; Lim et al., 2017; Atun et al., 2018; Subhashini et al., 2019). In folklore, DF is called “Banda” (Ghirtlahre et al., 2015) and “Vanda” (Khatoon et al., 2011), while DP is called “Benalu” (Elsyana et al., 2016). Additionally, DF and DP are also commonly known as Indian mistletoe and Indonesian mistletoe, respectively in Englishbecause their distribution is concentrated in India and Indonesia, respectively (Sinoriya et al., 2011; Lim et al., 2017; Alharits et al., 2020; Singh et al., 2020; Bhagat and Kondawara, 2021; Yapa et al., 2021). Moreover, they are rarely present in other countries like Thailand (Kwanda et al., 2013), China (Guo and Ren, 2019), and Bangladesh (Hasan et al., 2018). However, they can undermine economic crops such as *Mangifera indica* L., *Myristica fragrans* Houtt., and *Artocarpus heterophyllus* Lam. under infestation, often causing serious damage to local people (Kuramana et al., 2020; Sivaramakrishna et al., 2021; Yapa et al., 2021). In general, hemiparasites are generally considered as pests, and the locals often remove them from their hosts through chemical treatment and pruning (Wang et al., 2022).

Herein, relevant references covering from 1961 to 2022 were retrieved from Google Scholar, Sci-Finder, PubMed, Web of Science, Wiley, Elsevier, and SpringerLink. The search terms were “*Dendrophthoe*” and the subject was limited to medicinal value. Although 137 species names for the genus *Dendrophthoe* have been recorded in “The Plant List” (www.theplantlist.org), including 59 accepted names, 29 synonyms, and 49 unassessed species, species with pharmacological properties were focused on DF and DP. Accordingly, we reviewed their research results with respect to traditional medical uses, phytochemistry, pharmacology, toxicity, and applications, which paved the way for relevant researchers.

## 2 Traditional medical uses

DF and DP are traditional medicinal plants that are handed down in and around India and Indonesia. The traditional medical uses of DF and DP are used to treat cancer, wounds, ulcers, asthma, paralysis, fertility, skin diseases, menstrual disorders, and so on (Pattanayak et al., 2012; Sembiring et al., 2016; Rafe et al., 2017; Hardiyanti et al., 2018; Bhagat and Kondawar, 2019; Yismairai et al., 2019). Their extensive therapeutic effects on human diseases have allowed the locals to record them in the Ayurvedic system of medicine and other ethnomedicinal systems (Bhagat and Kondawar, 2019; Pramestya et al., 2019). In general, they are often crushed and mixed with other ingredients to make edible tea, decoction or plaster to treat human diseases, while raw materials are fed to livestock to improve growth performance (Pattanayak and Mazumder, 2009a; Reddy et al., 2019; Barkah and Lokesh, 2020; Nigam, 2022). In folklore, DF and DP are given various trivial names due to cultural differences. Furthermore, the different therapeutic effects show certain geographical differences in ethnomedical systems. The brief traditional medical uses of DF and DP were compiled in Table 1.

**TABLE 1:** Traditional medical uses of DF and DP in certain regions.

Species	Parts	Regions	Traditional medical uses	References
DF	Whole plant, bark, leaves, stems, and fruits	India	Treat rheumatic complaints, leucorrhoea, contraceptive, bone fracture, asthma, menstrual disorders, wound healing, and schizophrenia	Khatoon et al. (2011), Madhuri et al. (2013), Jain and Sharma (2016)
DF	Whole plant	India	Treat cooling, bitter, astringent, aphrodisiac, narcotic, diuretic, fertility, and cancer	Bhagat and Kondawar (2019), Reddy et al. (2019)
DF	Leaves, stems, and flowers	India	Treatment of wounds, mania, menstrual troubles, breathing problems, psychic disorders, and pulmonary tuberculosis	Pattanayak et al. (2012), Bhagat and Kondawara (2021)
DF	Whole plant	Bangladesh	Treatment of asthma, wounds, ulcer, pulmonary tuberculosis, and used as aphrodisiac, diuretic, astringent, and narcotic agents	Rafe et al. (2017)
DF	Whole plant	Sri Lankan	Treat cancerous tumors	Karunaratne and Uduwela (2020)
DF	Whole plant	Ayurveda	Treat diarrhea, swelling, renal calculi, and epilepsy	Shahare and Bodele (2019)
DP	Whole plant	Indonesia	Treat skin diseases, small sores, coughs, after childbirth, and hypertension	Elsyana et al. (2016), Pramestya et al. (2019)
DP	Whole plant	Kalimantan and Sulawesi	Treatment of diabetes and tonsillitis	Alharits et al. (2019)
DP	Whole plant	Thailand	Treatment of high blood pressure, chronic diseases, and immunological disorders	Kwanda et al. (2013)

The ethnomedical uses of DF are mainly documented in the Indian medical system. For example, DF is known as “Vanda”, “Vrksadani”, and “Vrksaruha” in the Indian Ayurvedic system of medicine, which is recorded to possess therapeutic effects on rheumatic complaints, leucorrhoea, contraception, bone fracture, asthma, menstrual disorders, wound healing, and schizophrenia (Khatoon et al., 2011; Madhuri et al., 2013; Jain and Sharma, 2016). The whole DF is used to treat cooling, bitter, astringent, aphrodisiac, narcotic, fertility, cancer, and diuretic in indigenous medicine (Bhagat and Kondawar, 2019; Reddy et al., 2019). Also, the whole DF is used as a cooling, astringent, aphrodisiac, narcotic, and diuretic agent in the traditional Indian system of medicine (Shalini et al., 2019a). Additionally, the ethnomedical properties of DF are documented in some countries. DF is called “Banda” by the tribals of India, and its leaves, stems, and flowers can be used to treat wounds, mania, menstrual troubles, breathing problems, psychic disorders, and pulmonary tuberculosis (Pattanayak et al., 2012; Bhagat and Kondawara, 2021). DF is also called “Farolla” in India and Bangladesh, which can be used for the treatment of asthma, wounds, ulcers, and pulmonary tuberculosis, as well as in formulations that are used as aphrodisiac, diuretic, astringent, and narcotic traditional medicines (Rafe et al., 2017). In other areas, DF is called “Pilla” in Sri Lankan, which can be used to treat cancerous tumors in the Sri Lankan ethnomedicinal system (Karunaratne and Uduwela, 2020). In Ayurveda, DF is known as “Vanda” or “Bandaka” and can be used to treat diarrhea, swelling, renal calculi, and epilepsy (Shahare and Bodele, 2019).

Ethnomedicinal surveys of DP are relatively few and are mainly reported in Indonesia. DP is commonly known as “Benalu” in Indonesiaand is used to treat skin diseases, small sores, coughs, after childbirth, and hypertension (Elsyana et al., 2016; Pramestya et al., 2019). In Sulawesi, DP can be used to treat mumps and cancer (Hasan et al., 2018; Yismairai et al., 2019). In Central Kalimantan and dyspnea in Southeast Sulawesi, DP can be used for the treatment of diabetes and tonsillitis (Alharits et al., 2019). In some areas of Thailand, DP can be used for the treatment of high blood pressure, chronic diseases, and immunological disorders (Kwanda et al., 2013).

## 3 Phytochemistry

**FIGURE 2:**
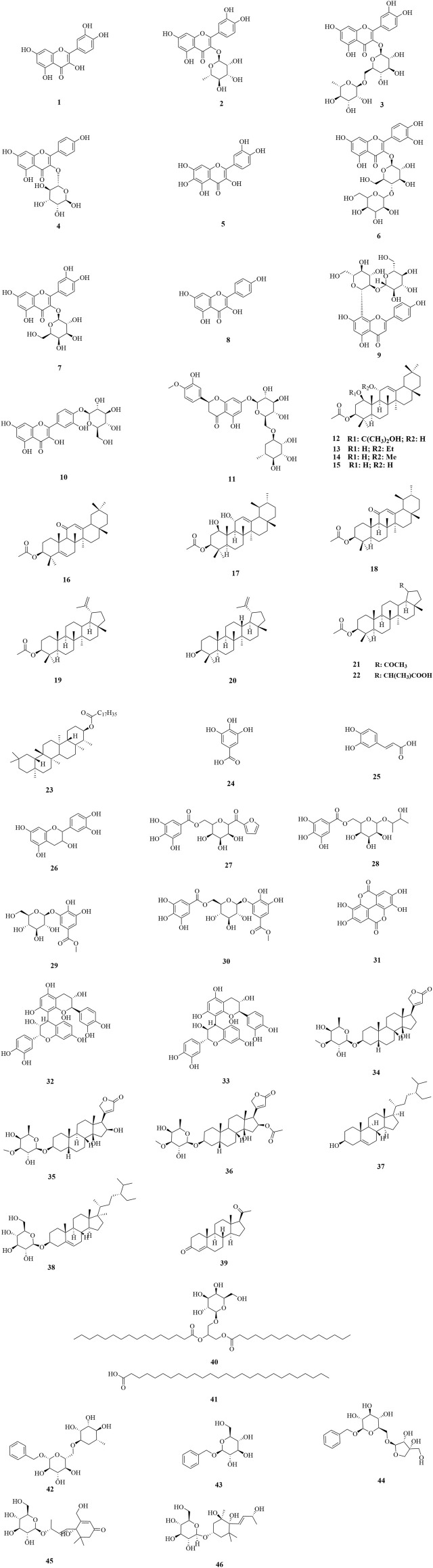
Chemical structures isolated from DF and DP.

Up to now, 46 compounds have been separated from DF and DP, including flavonoids (**1**–**11**), triterpenes (**12**–**23**), tannins (**24**–**33**), steroids (**34**–**39**), open-chain aliphatics (**40**–**41**), benzyl derivates (**42**–**44**), and cyclic chain derivatives (**45**–**46**). The names, chemical formulas, and masses of the 46 compounds were summarized in Table 2. Their chemical structures have been elucidated by NMR, MS, IR, HPLC, and UV methods, along with the comparison with the published data (Figure 2). Previous phytochemistry screening of DF and DP have reported the presence of alkaloids, anthraquinones, and coumarins (Asadujjaman et al., 2013; Fitrilia et al., 2015; Kristiningrum et al., 2018; Alharits et al., 2019; Susilowati and Sari, 2020; Tioline et al., 2021; Dhobale and Rothe, 2022), however, their structures have not been confirmed by modern spectroscopic techniques. Moreover, previous pharmacological studies on DF and DP have focused on the effects of crude extracts, organic fractions, and groups of phytochemicals, leading to limited bioactive compounds.

**TABLE 2:** Compounds isolated from DF and DP.

Num	Name	Formula	Mass	Species	Part	Hosts	References
**1**	Quercetin	C_15_H_10_O_7_	−	DP	Whole plant	*Averrhoa carambola* L. and *Ceiba pentandra* (L.) Gaertn	Rahmawati et al. (2014)
			−	DP	Leaves	*Averrhoa carambola* L	Artanti et al. (2006)
			−	DF	Whole plant	*Murraya koenigii* (L.) Spreng	Nair and Krishnakumary (1990)
			−	DF	Stems	−	Kachhawa et al. (2011a)
**2**	Quercitrin	C_21_H_20_O_11_	120 mg	DF	Fruits	*Shorea robusta* Gaertn	Mallavadhani et al. (2006)
			−	DF	Stems	−	Kachhawa et al. (2011a)
			−	DF	Whole plant	*Murraya koenigii* (L.) Spreng	Nair and Krishnakumary (1990)
			−	DP	Stems and leaves	*Annona squamosa* Linn	Nguyen et al. (2010)
			−	DP	Leaves	*Averrhoa carambola* L. and *Theobroma cacao* L	Artanti et al. (2006), Sembiring et al. (2022)
**3**	Rutin	C_27_H_30_O_16_	−	DF	Stems	−	Kachhawa et al. (2011a)
			−	DF	Whole plant	*Murraya koenigii* (L.) Spreng	Nair and Krishnakumary (1990)
			−	DP	Whole plant	*Averrhoa carambola* L. and *Ceiba pentandra* (L.) Gaertn	Rahmawati et al. (2014)
**4**	Kaempferol 3-*O*-*α*-L-rhamnopyranoside	C_20_H_18_O_11_	30 mg	DF	Fruits	*Shorea robusta* Gaertn	Mallavadhani et al. (2006)
			−	DP	Stems and leaves	*Annona squamosa* Linn	Nguyen et al. (2010)
**5**	Quercetagetin	C_15_H_10_O_8_	−	DF	Stems	−	Kachhawa et al. (2011a)
**6**	Meratin	C_27_H_30_O_17_	−	DF	Stems	−	Kachhawa et al. (2011a)
**7**	Hyperoside	C_21_H_20_O_12_	−	DF	Stems	−	Kachhawa et al. (2011a)
**8**	Kaempferol	C_15_H_10_O_6_	−	DF	Stems	−	Kachhawa et al. (2011a)
			−	DF	Whole plant	*Murraya koenigii* (L.) Spreng	Nair and Krishnakumary (1990)
			−	DF	Leaves	*Mangifera indica* L	Khatoon et al. (2011)
**9**	Apigenin-8-C-*β*-D (2″-*O*-*β*-D-glucopyranosyl)-glucopyranoside	C_27_H_30_O_15_	−	DF	Roots and leaves	−	Gangwar and Saxena (2010)
**10**	Quercetin 4′-*O*-*β*-glucopyranoside	C_21_H_20_O_12_	−	DP	Whole plant	*Averrhoa carambola* L. and *Ceiba pentandra* (L.) Gaertn	Rahmawati et al. (2014)
**11**	Hesperidin	C_29_H_36_O_14_	−	DP	Stems and leaves	*Annona squamosa* Linn	Nguyen et al. (2010)
**12**	3*β*-Acetoxy-1*β*-(2-hydroxy-2-propoxy)-11*α*-hydroxyolean-12-ene	C_35_H_58_O_5_	4 mg	DF	Fruits	*Shorea robusta* Gaertn	Mallavadhani et al. (2006)
**13**	3*β*-Acetoxy-11*α*-ethoxy-1*β*-hydroxy-olean-12-ene	C_34_H_56_O_4_	5 mg	DF	Fruits	*Shorea robusta* Gaertn	Mallavadhani et al. (2006)
**14**	3*β*-Acetoxy-1*β*-hydroxy-11*α*-methoxy-olean-12-ene	C_33_H_54_O_4_	12 mg	DF	Fruits	*Shorea robusta* Gaertn	Mallavadhani et al. (2006)
**15**	3*β*-Acetoxy-1*β*,11*α*-dihydroxy-olean-12-ene	C_32_H_52_O_4_	7 mg	DF	Fruits	*Shorea robusta* Gaertn	Mallavadhani et al. (2006)
**16**	3*β*-Acetoxy-12-ene-11-one	C_32_H_48_O_3_	−	DF	Leaves	−	Rafe et al. (2017)
**17**	3*β*-Acetoxy-1*β*,11*α*-dihydroxy-urs-12-ene	C_32_H_52_O_4_	22 mg	DF	Fruits	*Shorea robusta* Gaertn	Mallavadhani et al. (2006)
**18**	3*β*-Acetoxy-urs-12-ene-11-one	C_32_H_50_O_3_	16 mg	DF	Fruits	*Shorea robusta* Gaertn	Mallavadhani et al. (2006)
**19**	3*β*-Acetoxy-lup-20 (29)-ene	C_32_H_52_O_2_	190 mg	DF	Fruits	*Shorea robusta* Gaertn	Mallavadhani et al. (2006)
**20**	Lupeol	C_30_H_50_O	−	DF	Leaves	−	Rafe et al. (2017)
**21**	30-nor-lup-3*β*-Acetoxy-20-one	C_31_H_50_O_3_	42 mg	DF	Fruits	*Shorea robusta* Gaertn	Mallavadhani et al. (2006)
**22**	(20*S*)-3*β*-Acetoxy-lupan-29-oic acid	C_32_H_52_O_4_	150 mg	DF	Fruits	*Shorea robusta* Gaertn	Mallavadhani et al. (2006)
**23**	3-Friedelanol	C_48_H_86_O_2_	−	DP	Leaves and stems	*Annona squamosa* Linn	Nguyen et al. (2010)
**24**	Gallic acid	C_7_H_6_O_5_	99 mg	DF	Fruits	*Shorea robusta* Gaertn	Mallavadhani et al. (2006)
			−	DF	Leaves	*Mangifera indica* L	Khatoon et al. (2011)
			−	DP	Whole plant	*Averrhoa carambola* L. and *Ceiba pentandra* (L.) Gaertn	Rahmawati et al. (2014)
**25**	Caffeic acid	C_9_H_8_O_4_	−	DF	Leaves	*Mangifera indica* L	Khatoon et al. (2011)
**26**	Epicatechin	C_15_H_14_O_6_	−	DF	Leaves	*Mangifera indica* L	Khatoon et al. (2011)
			−	DP	Whole plant	*Averrhoa carambola* L. and *Ceiba pentandra* (L.) Gaertn	Rahmawati et al. (2014)
**27**	Scleropentaside F	C_18_H_18_O_11_	45.5 mg	DP	Whole plant	*Tectona grandis* L.f	Sahakitpichan et al. (2017)
**28**	Butane-2,3-diol-2-(6′-*O*-galloyl)-*O*-*β*-glucopyranoside	C_17_H_24_O_11_	6.3 mg	DP	Whole plant	*Tectona grandis* L.f	Sahakitpichan et al. (2017)
**29**	Methyl gallate 3-*O*-*β*-D-glucopyranoside	C_14_H_18_O_10_	212.3 mg	DP	Whole plant	*Tectona grandis* L.f	Sahakitpichan et al. (2017)
**30**	Methyl gallate 3-*O*-(6′-*O*-galloyl)-*β*-D-glucopyranoside	C_21_H_22_O_14_	422.3 mg	DP	Whole plant	*Tectona grandis* L.f	Sahakitpichan et al. (2017)
**31**	Ellagic acid	C_14_H_6_O_8_	−	DF	Leaves	*Mangifera indica* L	Khatoon et al. (2011)
**32**	Procyanidin B1	C_30_H_26_O_12_	18.5 mg	DP	Whole plant	*Tectona grandis* L.f	Sahakitpichan et al. (2017)
**33**	Procyanidin B3	C_30_H_26_O_12_	134.3 mg	DP	Whole plant	*Tectona grandis* L.f	Sahakitpichan et al. (2017)
**34**	Odoroside H	C_30_H_46_O_8_	−	DF	Leaves	*Nerium oleander* L	Boonsong and Wright (1961)
**35**	Strospeside	C_30_H_46_O_9_	−	DF	Leaves	*Nerium oleander* L	Boonsong and Wright (1961)
**36**	Neritaloside	C_32_H_48_O_10_	−	DF	Leaves	*Nerium oleander* L	Boonsong and Wright (1961)
**37**	*β*-Sitosterol	C_29_H_50_O	−	DP	Leaves	−	Soemardji et al. (2005)
			−	DF	Leaves	−	Rafe et al. (2017)
**38**	Daucosterol	C_35_H_60_O_6_	−	DP	Leaves and stems	*Annona squamosa* L	Nguyen et al. (2011)
**39**	Progesterone	C_21_H_30_O_2_	−	DP	Leaves	*Lansium domesticum* Corrêa	Mochamad et al. (2019)
**40**	Glycerol-1,2-dihexadecanoate-3-*O*-*β*-D-galactopyranose	C_41_H_78_O_10_	−	DP	Leaves and stems	*Annona squamosa* L	Nguyen et al. (2011)
**41**	Heptacosanoic acid	C_27_H_54_O_2_	−	DP	Leaves and stems	*Annona squamosa* L	Nguyen et al. (2011)
**42**	Benzyl-*O*-*α*-L-rhamnopyranosyl-(1 → 6)-*β*-D-glucopyranoside	C_20_H_30_O_9_	141.3 mg	DP	Whole plant	*Tectona grandis* L.f	Sahakitpichan et al. (2017)
**43**	Benzyl-*O*-*β*-D-glucopyranoside	C_13_H_18_O_6_	196.3 mg	DP	Whole plant	*Tectona grandis* L.f	Sahakitpichan et al. (2017)
**44**	Benzyl-*O*-*β*-D-apiofuranosyl-(1 → 6)-*β*-D-glucopyranoside	C_18_H_26_O_10_	162.2 mg	DP	Whole plant	*Tectona grandis* L.f	Sahakitpichan et al. (2017)
**45**	Bridelionoside A	C_19_H_30_O_9_	40.1 mg	DP	Whole plant	*Tectona grandis* L.f	Sahakitpichan et al. (2017)
**46**	Kiwiionoside	C_19_H_34_O_9_	9.0 mg	DP	Whole plant	*Tectona grandis* L.f	Sahakitpichan et al. (2017)

Flavonoids are a common class of secondary metabolites present in plants, and they are generally in the form of flavonoid glycosides (Nijveldt et al., 2001; Yang et al., 2018). According to the flavonoids yielded from DF and DP, they can be classified as flavones and flavonols, which presented as basic skeletal nuclei of C6-C3-C6. Common flavonoids such as quercetin (**1**), quercitrin (**2**), rutin (**3**), and kaempferol 3-*O*-*α*-L-rhamnopyranoside (**4**) have been yielded from DF and DP. Among them, compound **1** was previously yielded from DF (Nair and Krishnakumary, 1990; Kachhawa J. et al., 2011) and DP (Artanti et al., 2006; Rahmawati et al., 2014). Compounds **2**, **3**, and **4** were all previously isolated from DF (Nair and Krishnakumary, 1990; Mallavadhani et al., 2006; Kachhawa J. et al., 2011) and DP (Artanti et al., 2006; Nguyen et al., 2010; Rahmawati et al., 2014; Sembiring et al., 2022). Furthermore, quercetagetin (**5**), meratin (**6**), hyperoside (**7**), kaempferol (**8**), and apigenin-8-C-*β*-D-(2″-*O*-*β*-D-glucopyranosyl)-glucopyranoside (**9**), were previously isolated only from DF (Nair and Krishnakumary, 1990; Gangwar and Saxena, 2010; Kachhawa J. et al., 2011; Khatoon et al., 2011). Quercetin 4′-*O*-*β*-glucopyranoside (**10**) and hesperidin (**11**) were previously separated only from DP (Nguyen et al., 2010; Rahmawati et al., 2014).

Triterpenes consisting of six isoprene units are present in plants both individually and as glycosides, with tetracyclic triterpenes and pentacyclic triterpenes being the most common (Hill and Connolly, 2011). Notably, the triterpenes currently yielded from DF and DP are all pentacyclic triterpenes. Among them, except for 3-friedelanol (**23**), which was isolated from DP (Nguyen et al., 2010), the remaining 11 compounds were yielded from DF (Mallavadhani et al., 2006; Rafe et al., 2017), including 3*β*-acetoxy-1*β*-(2-hydroxy-2-propoxy)-11*α*-hydroxyolean-12-ene (**12**), 3*β*-acetoxy-11*α*-ethoxy-1*β*-hydroxy-olean-12-ene (**13**), 3*β*-acetoxy-1*β*-hydroxy-11*α*-methoxy-olean-12-ene (**14**), 3*β*-acetoxy-1*β*,11*α*-dihydroxy-olean-12-ene (**15**), 3*β*-acetoxy-12-ene-11-one (**16**), 3*β*-acetoxy-1*β*,11*α*-dihydroxy-urs-12-ene (**17**), 3*β*-acetoxy-urs-12-ene-11-one (**18**), 3*β*-acetoxy-lup-20 (29)-ene (**19**), lupeol (**20**), 30-nor-lup-3*β*-acetoxy-20-one (**21**), (20*S*)-3*β*-acetoxy-lupan-29-oic acid (**22**).

Tannins, a distinct taxonomic group of phenolic compounds with molecular weights ranging from 500 Da to 3,000 Da, are abundant in almost all plant foods and beverages (Serrano et al., 2009). They can be classified as hydrolyzable tannins, condensed tannins, and complex tannins, based on the different phenolic metabolites (Khanbabaee and Van Ree, 2001; Arbenz and Averous, 2015). Gallic acid (**24**), a common phenol, was previously isolated from DF (Mallavadhani et al., 2006; Khatoon et al., 2011) and DP (Rahmawati et al., 2014), while epicatechin (**26**) was previously reported from DF (Khatoon et al., 2011) and DP (Rahmawati et al., 2014) Moreover, scleropentaside F (**27**), butane-2,3-diol-2-(6′-*O*-galloyl)-*O*-*β*-glucopyranoside (**28**), methyl gallate 3-*O*-*β*-D-glucopyranoside (**29**), methyl gallate 3-*O*-(6′-*O*-galloyl)-*β*-D-glucopyranoside (**30**), procyanidin B1 (**32**), procyanidin B3 (**33**) were previously yielded from DP (Sahakitpichan et al., 2017). In addition, caffeic acid (**25**) and ellagic acid (**31**) were previously separated from DF (Khatoon et al., 2011).

Steroids are a class of natural compounds with a parent nucleus of cyclopentane-poly hydro phenanthrene, a trans-B/C ring, and a hydroxyl at the C-14 position (Riad et al., 2002; Cole et al., 2019; Ermolenko et al., 2020). According to the different substitutes at the C-17 position, cardiac glycosides, phytosterols, and C_21_-steroids have been confirmed from the yielded compounds of DF and DP. Previous phytochemical investigations have reported odoroside H (**34**), strospeside (**35**), and neritaloside (**36**) from DF (Boonsong and Wright, 1961), along with daucosterol (**38**) and progesterone (**39**) from DP (Mochamad et al., 2019). Additionally, *β*-sitosterol (**37**) was previously yielded from DF (Rafe et al., 2017) and DP (Soemardji et al., 2005).

For others, compounds **40–46** were all isolated from DP (Nguyen et al., 2011; Sahakitpichan et al., 2017). Among them, glycerol-1,2-dihexadecanoate-3-*O*-*β*-D-galactopyranose (**40**) and heptacosanoic acid (**41**) are open-chain aliphatics; benzyl-*O*-*α*-L-rhamnopyranosyl-(1 → 6)-*β*-D-glucopyranoside (**42**), benzyl-*O*-*β*-D-glucopyranoside (**43**) and benzyl-*O*-*β*-D-apiofuranosyl-(1 → 6)-*β*-D-glucopyranoside (**44**) are benzyl derivates; and bridelionoside A (**45**) and kiwiionoside (**46**) are cyclic chain derivatives.

## 4 Pharmacology

Previous pharmacological studies of DF and DP have revealed potential therapeutic possibilities for numerous ailments. First of all, anti-tumor and anti-microbial activities have been unveiled *in vitro* assays. On the basis of *in vitro* assays, several *in vivo* assays have found pharmacological properties of DF and DP against tumors, depression, fertility disorders, inflammatory responses, and others. Non-etheless, the pharmacological activities of DF and DP were superficially studied because findings regarding active molecules, relevant action mechanisms, along with clinical trials in humans were absent, which means that further investigations are indispensable to address these issues.

### 4.1 *In vitro* assays

#### 4.1.1 Anti-tumor activity

*In vitro* assays indicated that several extracts of DF or DP possess certain cytotoxic effects on breast cancer cells, Ehrlich ascitic carcinoma cells, skin cancer cells, chronic myeloid leukemia cells, and myeloma culture cells. To start with, MCF-7 cells were considered as a primary breast cancer model, and representative studies were listed below. The methanol extract of DP leaves was able to exert antiproliferative properties (0.04 μg/mL–100 μg/mL) with an IC_50_ value of 10.65 μg/mL, which might be attributed to the increased p53 and BAX, along with the decreased BCL-2 (Zainuddin et al., 2018). The ethyl acetate fraction of DP leaves growing on *Lansium parasiticum* (Osbeck) K.C.Sahni & Bennet (7.8 ppm–1,000 ppm) exhibited certain inhibitory activity with an IC_50_ value of 4.72 μg/mL (Yee et al., 2017). The aqueous extract of DF stems (62.5 μg/mL–500 μg/mL) exhibited higher inhibitory effects than ethanolic extract through MTT and SRB (sulforhodamine B) assays with IC_50_ values of 90 and 98 μg/mL, respectively (Dashora et al., 2011c). Furthermore, T47D cells were tested by the *n*-hexane fraction of DP, resulting in an IC_50_ value of 39.57 μg/mL (Subandrate et al., 2019). MCM-B2 cells were subjected to an *n*-hexane fraction of DP leaves growing on *Annona squamosa* L. (125 μg/mL), leading to an inhibitory activity of 41.5% (Elsyana et al., 2016). Additionally, the methanolic extract of DP enabled the K562 cells and the MCF-7 cells to arrest in G2/M and G1/S, respectively (Zamani et al., 2016; Zainuddin et al., 2018).

In addition, Ehrlich ascitic carcinoma cells, HaCat cells, and K562 cells were tested in previous studies of DF and DP. A study revealed that aqueous and ethanolic extracts (400 mg/kg) of DF stems growing on *Mangifera indica* L. were able to inhibit the growth of Ehrlich ascitic carcinoma cells with weights of 2.72 g and 1.96 g, respectively (Dashora et al., 2011a). The methanol extracts of DF leaves growing on *Mangifera indica* L. (10 μg/mL–80 μg/mL) could inhibit the growth of HaCat cells by MTT and SRB assays with cytostatic rates of 90.00% and 86.96%, respectively (Bhagat and Kondawara, 2021). The *n*-hexane fraction of DP leaves was unveiled to have suppressive effects on K562 cells with an IC_50_ value of 38.69% (Elsyana et al., 2016). Using methylene blue solution, a diethyl ether extract of DP stem barks was tested on myeloma culture cells, resulting in antiproliferation activity at doses ranging from 11.0 g/mL to 44 g/mL (Lazuardi, 2008).

**FIGURE 3:**
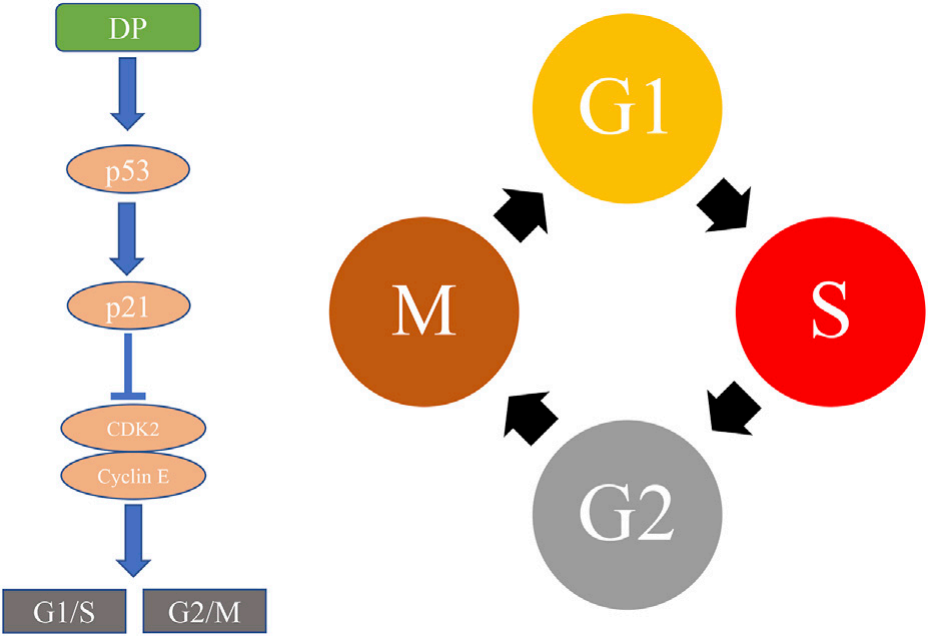
The underlying mechanism of cell cycle arrest of DP.

At present, significant advances have been made in the molecular mechanisms of cell cycle arrest by natural products that can inhibit cell cycle protein-dependent kinases and regulate proteinsused to control cell proliferation (Bailon-Moscoso et al., 2017). Bioactive molecules that target cell cycle protein-dependent kinases, in particular, have been used as new anti-cancer medicines. In general, p53 can regulate p21 which inhibits cell cycle protein-dependent kinase2 (CDK2) and cyclin E, as evidenced by the monitoring of G1/S and G2/M phase correction sites (Engeland 2018). Unfortunately, the number of protein-dependent stimulating enzymes is small in previous studies, therefore, more potential proteins need to be identified. The underlying mechanism of cell cycle arrest of DP was presented in Figure 3.

#### 4.1.2 Anti-microbial activity

In previous studies, the agar diffusion method was used to evaluate the anti-microbial activity by determining the diameter of the inhibition zone. For example, quercitrin isolated from DP leaves (1,000 μg/mL) was studied against *Escherichia coli*, *Salmonella typhi*, *Staphylococcus aeureus*, and *Pseudomonas* with inhibitory zones of 7.74 mm, 7.23 mm, 9.54 mm, and 8.82 mm, respectively (Hardiyanti et al., 2019c). Total flavonoids of DP leaves growing on *Polyalthia longifolia* (Sonn.) Thwaites (3%–9%) were deemed as potential antimicrobial agents against *Streptococcus mutans*, *Escherichia coli*, and *Candida albicans* with inhibitory zone diameters of 6 mm–17.25 mm, 3.55 mm–9.15 mm, and 4 mm–8.30 mm, respectively (Fahmi et al., 2018). Furthermore, the aqueous extract of DF was able to inhibit the proliferation of several strains. For instance, the aqueous extract of DF leaves parasitic on *Azadirachta indica* A.Juss. was able to attenuate *Staphylococcus aureus* with an inhibition zone of 10 mm (Kathayi and Nagarajan, 2018). The aqueous extract of DF twigs was demonstrated to exert antimicrobial activity against *Shigella flexneri*, *Staphylococcus epidermidis*, *Bacillus subtilis*, and *Escherichia coli* with inhibitory zones of 17 mm, 25 mm, 20 mm, and 22 mm, respectively (Shantipriya et al., 2015). For organic solvent extracts, the methanol and acetone extracts of the whole plant of DF (50 mg/kg) were found to inhibit *Shigella flexneri* by disc diffusion and agar well diffusion methods, with inhibition zones of 1.8 cm and 2.0 cm, respectively (Nayak et al., 2020).

A study found that the ethanol extract of aerial parts of DF against *Aspergillus fumigatus*, *Staphylococcus epidermidis*, *Salmonella typhi* with MIC values of 10 µg/disc (Pattanayak and Sunita, 2008). Unfortunately, few studies have reported the MICs and MLCs of the extracts of DF and DP and future studies should be carried out.

### 4.2 *In vivo* assays

#### 4.2.1 Oncotherapy

Active ingredients from DF or DP have been tested *in vivo* for their therapeutic effects on targeted tumors in mouse models induced by various chemicals. To start with, the therapeutic effects of DF extracts on breast tumors were evaluated using rats induced by dimethylbenzene [a]anthracene (DMBA). For example, a study found that the aqueous extract of DF leaves growing on *Shorea robusta* Gaertn. (400 mg/kg) was orally administrated to DMBA -induced rats, which reduced the tumor volume by 24.934%, showing more a significant therapeutic effect than chemical tamoxifen (Lobo et al., 2010). Another study revealed that the ethanol extract of the aerial part of DF was orally administrated to DMBA-induced rats (250 mg/kg–950 mg/kg), presenting normal control levels of liver indexes accompanied by an obvious decrease in breast tumors compared to the olive oil control group (Pattanayak S. P. and Mazumder P., 2011).

The mice induced by azoxymethane (AOM) were used to test the therapeutic effects of DP extracts. For instance, the ethanolic extract of DP was able to prevent colonic tumor damage. The oral administration of the ethanol extract of DP leaves growing on *Mangifera indica* L. (0.25 mg/g and 0.5 mg/g) was used for the treatment of mice induced by AOM, resulting in the colonic tissue with abnormal characteristics (Wicaksono and Permana, 2013). Moreover, the ethanolic extract of DF was found to release relevant cytokines and to arrest the cell cycle of colonic tumors. The mice induced by AOM were orally administrated with the ethanol extract of DP leaves growing on *Mangifera indica* L. (125 mg/kg–500 mg/kg), leading to a decrease in IL-22 and myeloperoxidase levels and an increase in the percentage of cells in the S phase compared with the model group (5% dextran sodium sulfate), which was correlated with p53 expression (Endharti et al., 2016).

#### 4.2.2 Therapeutics for depression

The oral administration of various extracts of DF or DP to rats or mice has been evaluated *via* several psychotropic tests, resulting in more active behavior than the diet without DF or DP. In the open field and hole cross tests, the ethanolic extract of DF stems grown on *Swietenia fabrilis* Salisb (200 mg/kg) inhibited locomotion in acetic acid-induced writhing models by 97.33% and 85.91%, respectively (Haque, 2016). In addition, the ethanol extract of DF leaves (250 mg/kg and 500 mg/kg) was orally administrated to mice, revealing a significant increase in the percentages of arms open, EPM arm open time, and motor coordination in the rotating bar test, and an increase in the percentage of time spent in the light box and the number of transitions in the light-dark box test, which was associated with a GABA(A) receptor-mediated mechanism of action (Aryal and Khan, 2015). The oral administration of ethanolic extract of DF leaves enabled the rats to exhibit potent anti-depression activity with average response times of immobility of 86.7 and 76.08, respectively, compared to the control group treated with the vehicle (Dubey et al., 2015). The gavage of the ethanolic extract of DF leaves (200 mg/kg and 400 mg/kg) significantly increased motor activity and resting time and decreased depression in the forced swimming experiment and the tail suspension experiment in mice compared with the vehicle-treated group (Logesh Kumar et al., 2019).

#### 4.2.3 Effects on fertility

Kaempferol, isolated from the stems of DF, was orally administrated to rats at 50 mg/rat/day for 60 days, manifesting low sperm motility and density as well as a marked reduction in serum testosterone levels than the normal control group (Kachhawa J. B. et al., 2011). Several extracts, in particular, have been demonstrated to possess potential therapeutic effects on fertility disorders. In terms of methanolic extract of DF or DP, follicle-stimulating hormone (FSH) and progesterone levels in female rats were tested by the oral administration of the methanolic extract of DP leaves (100 mg/kg) using the random evidence investigator analyzer, showing values of 9.28 miu/mL ± 6.72 miu/mL and 33.55 nmol/L ± 13.96 nmol/L, respectively (Mochamad and Hermanto, 2014). The rats were orally administrated with the ethanol extract of the aerial parts of DF (250 mg/kg–950 mg/kg), revealing anti-implantation activity, based on a decrease in the number of litters delivered after postcoital testing (Pattanayak and Mazumder, 2009b). Additionally, the oral administration of the methanolic extract of DF stems at 200 mg/rat/day could reduce the weights of reproductive organs, sperm count, and sperm motility in male rats compared to the vehicle-treated control group, leading to sterile state (Gupta and Kachhawa, 2008). The methanol extract of DF stems was orally administrated to male rats at 200 mg/rat/day, resulting in a reduction of sperm count and sperm motility, along with a decrease in the content of total protein, sialic acid, and glycogen in the testicles than the standard drug group [lonidamine(1-(2,4-dichlorobenzyl)-1H-indazole-3carboxylic acid] (Gupta and Kachhawa, 2007). The oral administration of methanol extract of DF stems (200 mg/kg) resulted in decreased sperm motility, density, and serum testosterone levels, and the degenerative changes in the varicocele in male albino rats compared with the untreated control group (Gupta and Kachhawa, 2007). Furthermore, the oral administration of chloroform fraction of DF stems at 50 mg/day revealed the suppression of sperm density and motility in the epididymal tail, accompanied by decreased protein and sialic acid in the testis and cauda epididymis, as well as increased testicular cholesterol compared to the untreated group (Kachhawa J. B. et al., 2011).

#### 4.2.4 Therapeutics for inflammatory responses

The paw edema in rats or mice induced by chemicals was considered as a representative inflammatory model. For instance, the instillation of ethanolic extract of DP leaves grown on *Mangifera indica* L. (600 mg/kg) to 2,4,6-trinitrobenzenesulfonic acid-induced mice contributed to attenuating colon shortening and myeloperoxidase, along with significant improvement in colitis compared to the control group (50% ethanol-phosphate-buffered saline) (Endharti and Permana, 2017). For paw edema in rats induced by carrageenan, the oral administration of aqueous and methanolic extracts of DF leaves growing on *Mangifera indica* L. (300 mg/kg) resulted in inhibitory indices of 30.95% and 23.41%, respectively, and reduced the formation of granuloma tissue by 51% and 48%, respectively (Patil et al., 2011).

Similar results were also presented by the treatment of fractions with diverse polarities. The gavage of chloroform fraction of DF stems growing on *Swietenia fabrilis* Salisb. (200 mg/kg) enabled the inhibition rate of the carrageenan-induced paw edema mice to reach 90.24% (Haque, 2016). The ethyl acetate fraction of the whole plant of DP (2000 mg/kg) was orally administrated to rats induced by carrageenan, resulting in an inhibition of 41.46%, which might be ascribed to the presence of quercetin (Mustarichie et al., 2015). The gavage of petroleum ether fraction of DF leaves growing on *Swietenia fabrilis* Salisb. (200 mg/kg) was found to exert pharmacological activity against paw edema in mice induced by carrageenan with an inhibitory volume of 90.24% (Haque et al., 2014c).

#### 4.2.5 Immunoregulation

Polysaccharides are considered to be the primary active ingredient of plants (Huang et al., 2022; Li et al., 2022). Currently, polysaccharides of DF have been used as dietary supplements. Polysaccharides extracted from DF leaves growing on *Azadirachta indica* A.Juss., for example, were fed to fish as a supplemented diet (1%), and the results showed an increase in lysozyme and TNF-α expression, as well as an increase in relative survival percentage compared to the positive control group (0.1% MacroGard™) (Shalini et al., 2019a). The tilapias were fed polysaccharides isolated from DF leaves growing on *Azadirachta indica* A.Juss. (200 mg/kg), resulting in a reduction in the percentage of mortality resulting from *Aeromonas hydrophila* infections and upregulation of IL-1β and lysozyme genes compared with the control group (MacroGard™, 20 mg/kg) (Shalini et al., 2019b).

Additionally, the oral administration of ethanolic extract of DF or DP enabled the mice to improve their immune systems. For example, a study found that the ethanol extract of DP leaves growing on *Mangifera indica* L. (150 mg/kg–600 mg/kg) was able to increase IL-2 levels and the percentage of CD4^+^CD28^+^ and CD8^+^CD28^+^ in aged female mice compared to the control group (phosphate buffer saline) (Handono et al., 2021). The ethanol extract of the aerial parts of DF parasitic on *Azadirachta indica* A.Juss (250 mg/kg–950 mg/kg) was employed in female rats, revealing a significant increase in NO production by peritoneal macrophages and phagocytic activity of polymorphonuclear cells and the reticuloendothelial system compared with the untreated group (Pattanayak S. P. and Mazumder P. M., 2011). The pro-inflammatory indices mentioned above, such as IL-1β, TNF-α, and IL-2, are involved in innate immunity, which can enhance the expression of non-specific immune mechanisms and immune-related genes, revealing a potential link between inflammatory response and immunity.

#### 4.2.6 Therapeutics for hyperglycemia and hypertension

Alloxan-induced rats were commonly considered diabetic models and have been used to evaluate the therapeutic effects of several extracts. A study found that alloxan-induced rats gavaged with the methanol extract of DF stems growing on *Mangifera indica* L. (200 mg/kg) could result in decreased levels of blood cholesterol and triglyceride, which might indicate that DF was possible to possess a therapeutic effect on type-II diabetes mellitus (Anarthe et al., 2008). In addition, the oral administration of the methanol extract of DP leaves (50 mg/kg and 400 mg/kg) enabled alloxan-induced rats to have significant antidiabetic activity with a notable oral glucose tolerance equivalent to the antihyperglycemic metformin (Hasan et al., 2018).

Active ingredients of DP were found to have protective and regulatory effects on arterial vasculature. A study pointed out that the hypertensive rats gavaged with the methanol extract of DP leaves growing on the *Mangifera indica* L. (200 mg/kg) reduced the amount of cellular necrosis and altered the width of the white matter areas of the brain in hypertensive rats treated with deoxycorticosterone acetate salt (Saputri et al., 2021).

#### 4.2.7 Therapeutics for other ailments

DF and DP were also used for attenuating hypercholesteremia, promoting urination, treating diarrhea, protecting the liver, and retarding oxidation. Despite the number of studies involving in the above therapeutic properties was limited, these findings also indicated certain pharmacotherapeutic possibilities that need further investigation. The gavage of water extract of DP leaves (800 mg/kg) was able to reduce the total cholesterol levels and improve liver histopathology in mice treated with a hypercholesterolemic diet (Mu’nisa et al., 2019). The oral administration of the petroleum ether fraction of DF leaves (250 mg/kg and 500 mg/kg) resulted in higher urine output compared with furosemide in the control group (Reddy et al., 2019). The castor oil-induced mice were gavaged with the ethanol extract of DF leaves growing on *Swietenia fabrilis* Salisb. (200 mg/kg), showing a reduction in the severity and frequency of diarrheal pairs with an inhibitory rate of 83.47% (Haque et al., 2014b). The CCl_4_-induced mice were orally administrated with water and ethanol extracts of DF leaves growing on *Swietenia fabrilis* Salisb. (200 mg/kg), leading to increased activities of liver markers, including transaminase (AST), alanine transaminase (ALT), alkaline phosphatase (ALP), total protein, and bilirubin (Haque et al., 2014a). An assay revealed that the gavage of ethanolic extract of the aerial part of DF (250 mg/kg and 500 mg/kg) could exert anti-oxidant effects in excisional and incisional wound models in rats through the inhibition of lipid peroxidation, the decrease of glutathione and superoxide dismutase, along with the increase of catalase activity (Pattanayak and Sunita, 2008). The above results of *in vivo* studies of DF and DP were summarized in Table 3.

**TABLE 3:** Results of *in vivo* studies of DF and DP.

Pharmacological properties	Species	Hosts	Parts	Extracts/Compounds	References
Oncotherapy	DF	*Shorea robusta* Gaertn	Leaves	Aqueous extract	Lobo et al. (2010)
	DF	−	Aerial part	Ethanol extract	Pattanayak and Mazumder (2011a)
	DP	*Mangifera indica* L	Leaves	Ethanol extract	Wicaksono and Permana (2013)
	DP	*Mangifera indica* L	Leaves	Ethanol extract	Endharti et al. (2016)
Therapeutics for depression	DF	*Swietenia fabrilis* Salisb	Stems	Ethanol extract	Haque (2016)
	DF	−	Leaves	Ethanol extract	Aryal and Khan (2015)
	DF	*Mangifera indica* L	Leaves	Ethanol extract	Dubey et al. (2015)
		*Psidium guajava* L			
	DF	−	Leaves	Ethanol extract	Logesh Kumar et al. (2019)
Effects on fertility	DF	−	Stems	Kaempferol	Kachhawa et al. (2011b)
	DP	−	Leaves	Methanolic extract	Mochamad and Hermanto (2014)
	DF	−	Aerial parts	Ethanol extract	Pattanayak and Mazumder (2009b)
	DF	−	Stems	Methanolic extract	Gupta and Kachhawa (2008)
	DF	−	Stems	Methanol extract	Gupta and Kachhawa (2007)
	DF	−	Stems	Methanol extract	Gupta and Kachhawa (2007)
	DF	−	Stems	Chloroform fraction	Kachhawa et al. (2011b)
Therapeutics for inflammatory responses	DP	*Mangifera indica* L	Leaves	Ethanol extract	Endharti and Permana (2017)
	DF	*Mangifera indica* L	Leaves	Aqueous and methanolic extracts	Patil et al. (2011)
	DF	*Swietenia fabrilis* Salisb	Stems	Chloroform fraction	Haque (2016)
	DP		Whole plant	Ethyl acetate fraction	Mustarichie et al. (2015)
	DF	*Swietenia fabrilis* Salisb	Leaves	Petroleum ether fraction	Haque et al. (2014c)
Immunoregulation	DF	*Azadirachta indica* A.Juss	Leaves	Polysaccharides	Shalini et al. (2019a)
	DF	*Azadirachta indica* A.Juss	Leaves	Polysaccharides	Shalini et al. (2019b)
	DP	*Mangifera indica* L	Leaves	Ethanol extract	Handono et al. (2021)
	DF	*Azadirachta indica* A.Juss	Aerial parts	Ethanol extract	Pattanayak and Mazumder (2011b)
Therapeutics for hyperglycemia and hypertension	DF	*Mangifera indica* L	Stems	Methanol extract	Anarthe et al. (2008)
	DP	*−*	Leaves	Methanol extract	Hasan et al. (2018)
	DP	*Mangifera indica* L	Leaves	Methanol extract	Saputri et al. (2021)
Therapeutics for other ailments	DP	*−*	Leaves	Water extract	Mu’nisa et al. (2019)
	DF	*−*	Leaves	Petroleum ether fraction	Reddy et al. (2019)
	DF	*Swietenia fabrilis* Salisb	Leaves	Ethanol extract	Haque et al. (2014b)
	DF	*Swietenia fabrilis* Salisb	Leaves	Water and ethanol extract	Haque et al. (2014a)
	DF	*−*	Aerial part	Ethanolic extract	Pattanayak and Sunita (2008)

## 5 Toxicity

Preliminary toxic tests for DF and DP were focused on oral toxicity in mice and brine shrimp lethality assays, showing relatively high LC_50_ or LD_50_ values with a wide range of safe doses in general. Additionally, the toxic effects on the physiological organ functions of rats were small, suggesting low toxicity to a certain extent.

### 5.1 Mouse oral toxicity test

Several toxic studies have reported with the oral administration of the ethanolic extract of DF to rats. An oral acute toxic test indicated that aqueous and ethanolic extracts of DF stems growing on *Mangifera indica* L. resulted in LD_50_ values of 4 g/kg (Dashora et al., 2011a). The rats subjected to the ethanolic extract of aerial parts of DF growing on *Azadirachta indica* A.Juss. were found to have an LD_50_ value of 1.75 g/kg (Pattanayak and Mazumder, 2010). The ethanol extract of aerial parts of DF parasitic on *Azadirachta indica* A.Juss. enabled the neurobehavioral rats to reach an LD_50_ value of 4.55 g/kg (Pattanayak and Mazumder, 2009a). Similar reports were also seen in DP. The ethanol extract of DP growing on *Mangifera indica* L. was employed to male and female rats, showing LD_50_ values of 34.28 g/kg and 22.41 g/kg, respectively (Nurfaat, 2016). The oral administration of aqueous and ethanolic extracts of DP to rats revealed LD_50_ values of 17.78 g/kg and 12.59 g/kg, respectively (Mustarichie et al., 2016).

In addition to the above results, several studies have found neither lethality nor significant toxic effects following the administration of high doses of extracts to rats. For instance, the oral administration of the ethanol extract of DF leaves to rats showed neither abnormal effects nor moribund stages (Aryal and Khan, 2015). Aqueous and ethanolic extracts of DF leaves growing on *Swietenia fabrilis* Salisb. were orally administrated to rats at doses of 200 mg/kg–3,200 mg/kg, resulting in no obvious changes in internal organs compared to the normal group (Haque et al., 2014a). The ethanolic extract of DF barks was employed on rats, resulting in no lethality occurrence even if the doses were upgraded to 2,000 mg/kg (Pattanayak et al., 2008). Similarly, *β*-sitosterol and quercitrin isolated from DP leaves were demonstrated to be non-toxic, with lethality up to 2,000 mg/kg (Soemardji et al., 2005).

### 5.2 Brine shrimp lethality test

The brine shrimp lethality test (BSLT) with convenient and sensitive characteristics was designed to preliminarily evaluate the toxic effects of DF and DP and the results are presented as LC_50_ values in general. Compared with the oral toxic effects in mice, brine shrimp tend to have lower LC_50_ values than mice due to the sensitivity of the extracts. Moreover, DP tends to show a higher LC_50_ value than DF, based on current results.

Most of the DP studies have been undertaken to investigate the toxicity of organic fractions and extracts. For instance, the *n*-hexane fraction of DP leaves was found to possess toxic effects on shrimp larvae with an LC_50_ value of 41.59 ppm (Hardiyanti and Marpaung, 2018). The *n*-hexane fraction of DP leaves growing on clove was revealed to have an LC_50_ value of 55.31 μg/mL by BSLT (Elsyana et al., 2016). For organic extracts, the methanolic extract yielded from DP leaves was conducted by BSLT, resulting in an LC_50_ value of 13.95 μg/mL (Hardiyanti et al., 2019a). Additionally, similar results were also found in phytochemicals. An *in silico* approach revealed that quercitrin isolated from DP possessed a harmless LD_50_, but long-term use was not suggested (Sjakoer et al., 2021). The total flavonoids of DP leaves growing on *Polyalthia longifolia* (Sonn.) Thwaites were found to have an LC_50_ value of 30.06 mg/L by BLST (Fahmi et al., 2018).

Some studies have also reported the toxicity of DF. The methanol, ethyl acetate, and *n*-hexane extracts of DF leaves were investigated by BSLT, resulting in LC_50_ values of 38.815 μg/mL, 54.827 μg/mL, and 38.815 μg/mL, respectively (Runtuwene and Kamu, 2020). The aqueous and ethanolic extracts of stems of DF were investigated by BLST, displaying LC_50_ values of 90 and 120 μg/mL, respectively (Dashora et al., 2011a). The ethanolic extract of DF leaves was found to possess an LC_50_ value of 100 μg/mL by BLST (Hasan et al., 2006).

## 6 Applications

### 6.1 Pharmaceutical preparations

DF can be used as raw materials to manufacture tablets with higher absorption and stability than traditional preparation methods. For instance, the mucilage of DF as a supplementary material at a concentration of 6% could be prepared as paracetamol granules using the wet granulation technique, which possessed a friability of 0.98%–0.53%, a disintegration time of 10 min–17 min, and a dissolution of more than 9% in 70 min (Kothawade et al., 2010; Lazuardi et al., 2021). The twigs of DF could be processed into matrix tablets of astemizole with better homogeneity in terms of weight and content than tablets without DF, which might indicate that they are potential natural materials to make sustained-release matrix tablets (Bhoyar et al., 2012).

The active ingredients of DF could be processed into nanoparticles that were conducive to facilitating their metabolism, absorption, distribution, and excretion, which might provide a novel insight into the pharmacokinetics. Quercetin isolated from DF was formulated by spontaneous emulsification with oil, Tween 80, and phosphate bufferto yield quercetin nanoemulsions with an average particle size ranging from 10.2 nm to 36.5 nm and a polydispersity index of less than 0.5 (Atun et al., 2020). The aqueous extract of DF as a reducing and stabilizing agent could be processed into silver nanoparticles having cytotoxic effects on MCF-7 cells by optimizing various reaction conditions (Sathishkumar et al., 2014).

### 6.2 Combined medication

DP combined with common clinical drugs was found to produce a synergistic effect on certain conditionsand exhibited more significant therapeutic effects compared to individual use. For example, the ethanolic extract of DP combined with doxorubicin could decrease intracellular calcium concentration and surviving levels and increase the number of apoptotic MCF-7 cells, which exhibited more significant cytotoxic effects than doxorubicin alone (Endharti et al., 2018b). Compared with the 5-fluorouracil only group, the combination of ethanolic extract of DP and 5-fluorouracil more significantly decreased the survival of HeLa cells by increasing the percentage of p21 and apoptosis, showing a synergistic effect to restrain the proliferation of tumor cells (Endharti et al., 2018a). The ethanolic extract nanoparticles of DP together with cisplatin could produce a synergistic effect on HeLa cells, which possessed more significant cytotoxic effects than individual medicines (Mutiah et al., 2018). Furthermore, some studies have reported that combined medication might further extend the therapeutic effects of DF and DP. An assay indicated that methanolic extracts of DP and *Scurrula. atropurpurea* (Bl.) Dans were able to exert renal reinforcement by reducing the levels of creatinine, urea nitrogen, urea, and renal cell necrosis in rats, which further enlarged the pharmacotherapeutic range of DP (Anjani et al., 2021). The ethanolic extract of DP paired with paclitaxel was able to attenuate the growth of MCF-7 cells by reducing the expression of tubulin βIII and microtubule-associated protein 4, which might provide a promising method for the treatment of breast cancer (Permana et al., 2021).

With the development of medical plants combined with modern technology, potential active molecules have been revealed in medicine made from two or more plants. For example, bioactive molecules including flavonol, kaempferol, casticin, quercetin, quercitrin, and isoquercitrin were yielded from DP and *Scurrula atropurpurea* (Blume) Danser *via* molecular-specific docking analysis, and found to possess suppressive effects on the angiotensin-converting enzyme responsible for hypertension (Sjakoer et al., 2021). Quercetin, kaempferol, and aloe-emodin produced from DP and *Cassia alata* L. were able to inhibit the SARS coronavirus major peptidase and the 3-chymotrypsin-like cysteine protease 3 enzyme using a computational approach, suggesting their potential development as candidates against COVID-19 (Ernawati et al., 2021).

## 7 Discussion

In terms of phytochemistry, the issue of unclear active ingredients and the insufficient number of 46 compounds in DF and DP has led to limited elucidation for pharmacological activities. In recent years, phytochemistry screening has undergone great changes with the emergence of methods characterized by rapid, trace, and large-scale application of a variety of advanced technological tools, including high-throughput screening, chemical and biological characterization (Alves-Silva et al., 2020; Blay et al., 2020; Le Basle et al., 2020). The application of high-throughput screening, GC-MS, LC-MS, NMR, chemometrics, and pattern recognition theory contributed to the simultaneous online separation and the acquisition of qualitative/quantitative data for multiple groups of compounds. In the future, the above techniques and theory should be applied in DF and DP research, which make it possible to elucidate the pharmacological basis of botanical drugs.

The traditional medical uses of DF and DP have shown extensive therapeutic possibilities for human ailments with regional differences. However, these uses were simply recorded in the relevant medical systems with the lack of preparation methods and instruction for specific clinical applications, which require refinement and clarification by conducting civilian surveys. It is worth noting that the treatment of ulcers, asthma, tuberculosis, rheumatic complaints, wounds, and paralysis for traditional uses has not yet been validated compared to pharmacological results in current research. Given traditional medical uses as a valuable reference for current research, which require us to ascertain unproven uses by performing relevant pharmacological experiments.

Although remarkable results have been achieved in verifying the efficacy of DF and DP through cellular and animal models, studies on their mechanisms have been rarely reported. In general, phytopharmaceuticals are characterized by their multi-component and multi-target properties, which tend to cause difficulties for researchers in elucidating their molecular mechanisms. With the development of chemistry, biology, and informatics, the integrated application of these disciplines is playing an increasingly important role in pharmacology. Notably, multidimensional association models based on “chemical fingerprint-metabolic”, “fingerprint-network”, “target-disease effect”, and “pharmacokinetic-pharmacodynamic” assays have become a hot topic of current research (Gallo, 2010; Nielsen et al., 2011; Katz and Baltz, 2016; Xiao et al., 2022). Through theories and methods such as metabolomics, proteomics, transcriptomics, phenomics, genomics database mining, and bioinformatics analysis, a solid foundation has been laid to elucidate the scientific content of phytopharmaceuticals, which should be promoted and applied in the future research of DF and DP.

In order to evaluate the safety of DF and DP, several toxic tests in mice and brine shrimp have been established. As a result, their various extracts appear to be safe, considering their high LC_50_ or LD_50_ values and the absence of significant adverse effects. However, these findings are emphasized in the preliminary assessment of individual lethality and physiological manifestations. Therefore, future toxicological studies on the mechanism of action and metabolic transformation should be conducted using isolated organs, cultured cells, or organelles. Recent findings have revealed new prospects in pharmaceutical preparations and combined medication for DF and DP. As we have seen, DF was deemed as an important raw material that could be processed into tablets and nanoparticles characterized by better absorption, metabolism, and distribution, providing new perspectives for pharmacokinetics. In addition, DP combined with classic drugs was found to exhibit stronger therapeutic effects compared to individual usage. However, there was a significant imbalance in the applications of DF and DP. As we have seen, the pharmaceutical preparation was concentrated on DF, while the combined medication was focused on DP. Perhaps, the pharmaceutical preparation for DP and the combined medication for DF need further investigation, which not only extends the scope of application but also contributes to explaining the relationship between DF and DP.

Modern molecular docking and network pharmacology techniques have emerged as convenient and efficient assays for identifying potential bioactive molecules and action targets, as a result of the integration of computer technology and medical plants. Notably, the applicability of these techniques is based on the presence of active molecules in plants, which also demonstrates the close link between phytochemistry and pharmacology. From a foresight perspective, what illuminates the research direction is the combination of pharmacology and phytochemistry with reference to traditional medical uses. Particularly, other *Dendrophthoe* species like *Dendrophthoe trigona* (Wt. & Arn.) Danser and *Dendrophthoe praelonga* (Blume) Miq. were also found to have certain biological activities (Joshi et al., 2012; Patel et al., 2012; Irfan et at., 2020), which need further studies to develop their medical value.

## 8 Conclusion

Despite the fact that DF and DP were considered as pests that can harm multiple hosts, their traditional uses for treating a variety of ailments such as cancer, ulcers, asthma, paralysis, and skin diseases, have allowed them to capture our attention. Current research has achieved remarkable advances in chemical constituents, pharmacological properties, safety, and applications for DF and DP. As we can see, phytochemical investigations have revealed the presence of 46 compounds from DF and DP, including flavonoids, triterpenes, tannins, steroids, open-chain aliphatics, benzyl derivates, and cyclic chain derivatives. Pharmacological studies have demonstrated therapeutic possibilities of DF and DP against tumors, depression, fertility disorders, inflammatory responses, and others. Toxicity tests suggested that active extracts of DF and DP possessed a wide range of safety without significant adverse effects. In addition, pharmaceutical preparation for DF and combined medication for DP indicated the great potential for medical applications. However, there are still many limitations that need to be addressed, and the following are some suggestions to address these issues.First, traditional medical uses of DF and DP were simply recorded in ethnomedical systems without specific introduction, which needs us to carry out ethnomedical surveys for further refinement and clarification. Second, the issue of unclear active molecules and insufficient compounds from DF and DP limits the elucidation of pharmacological activities, and the application of high-throughput screening, LC-MS, and GC-MS may contribute to discovering compounds. Third, pharmacological research of DF and DP has not been illustrated in molecular biology, leaving underlying mechanisms for corresponding diseases unclear. Therefore, common assays such as western blot, polymerase chain reactions, and metabolomics should be undertaken in future research. Forth, toxic tests of DF and DP were not rigorous, and the addition of cytotoxicity, genotoxicity, and clinical trial tests will make their safety assessment more comprehensive. Fifth, DF and DP focused on diverse applications, and their biochemical differences and relationship need further investigation.
